# Constipation and sleep behaviour disorder associate with processing speed and attention in males with Parkinson’s disease over five years follow-up

**DOI:** 10.1038/s41598-020-75800-4

**Published:** 2020-11-04

**Authors:** Wee Lee Kong, Yue Huang, Elizabeth Qian, Margaret J. Morris

**Affiliations:** 1 Department of Pharmacology, School of Medical Sciences, Faculty of Medicine, University of New South Wales, Sydney, NSW 2052 Australia; 2 China National Clinical Research Center for Neurological Diseases, Beijing Tiantan Hospital, Capital Medical University, Beijing, 100071 China

**Keywords:** Parkinson's disease, Constipation, Sleep disorders

## Abstract

Constipation and REM sleep behaviour disorder (RBD) are the earliest non-motor manifestations of Parkinson’s disease (PD). Among non-motor symptoms of PD, it is unclear whether constipation and RBD at early stages of PD are related to cognitive outcomes at later stages. Herein, this study aims to investigate whether the presence of constipation and RBD have an impact on future cognitive outcomes in PD. Access to Parkinson’s Progression Markers Initiative (PPMI) database of 360 PD patients with longitudinal observation was requested. Constipation, probable RBD (pRBD) and neuropsychological task scores of PD patients were assessed at baseline and after 5 years. Linear mixed-effects modelling, controlling for gender, age, years of education and LEDD was used to evaluate the association between baseline constipation, pRBD and cognitive performance on follow-up. Gender differences in neuropsychological test performances were found, with men having worse global cognition, speed-attention processing, verbal learning and memory than women at early stages of the disease. We found constipation and pRBD are strongly associated with future decline in some cognitive measures among PD patients, more prominently in men. Our data suggest that early assessment of pRBD and constipation may allow better understanding of the progression of cognitive changes in later phases of PD.

## Introduction

Parkinson’s disease (PD) is recognised as one of the most common neurodegenerative disorders, with advancing age being its greatest risk factor^1^. Worldwide, PD afflicts 10 million people, and this figure is expected to double by 2030 due to our ageing population. Currently, it afflicts 1–2% of the population over the age of 60^2^. Notably, epidemiological studies show that the risk of PD is 1.5 to 2 times greater in men than in women^1,3,4^.

Although PD has been classified as a movement disorder, a myriad of significant non-motor features can develop during the preclinical period, which become more prevalent with advancing disease^5,6^. These non-motor symptoms can antedate the onset of the prototypical motor symptoms by over a decade^7,8^. The causes of non-motor symptoms are multifactorial, involving neuronal loss in the dopaminergic, noradrenergic, cholinergic, and serotonergic systems. It is postulated that the manifestation of non-motor symptoms in PD is associated to the presence of Lewy bodies in specific non-nigral brainstem nuclei, which are responsible in fundamental homeostatic regulations, including gastrointestinal homeostasis, circadian rhythms and mood control^9^.

One of the most common and debilitating non-motor symptoms of PD is gastrointestinal dysfunction, which has a prevalence of 70–80%^10^. Gastrointestinal symptoms are identified as regurgitation, bloating, constipation, nausea, delayed gastric emptying, malnutrition and *Helicobacter pylori* infection^11^. Of all the gastrointestinal symptoms, constipation is recognised as the most prominent autonomic disturbance in preclinical PD as observed in 20% to 80% of PD patients^12^.

Recent evidence shows gastrointestinal dysfunction is present during the preclinical stages of PD, suggesting the PD pathological process could initiate in the gut^13,14^. Strong evidence supporting the presence of constipation during preclinical PD comes from the Honolulu Heart Program, that followed 6790 men over a 24-year period, which indicated that men with less than 1 bowel movement per day have a 4.1-fold risk of PD compared with men with 2 movements per day^15^. A recent preclinical study has highlighted constipation as an early feature of α-synucleinopathy which may precede the onset of motor symptoms in PD^16^. Although constipation has been investigated as a prodromal marker of PD^17^, the exact association between alterations in the gastrointestinal system and CNS in PD remains elusive.

According to the third edition of International Classification of Sleep Disorders (ICSD-3), a diagnosis of rapid eye movement sleep behaviour disorder (RBD) is a parasomnia, characterised by repeated episodes of sleep-related vocalisation and/or complex motor behaviours. These behaviours are documented by polysomnographic recordings, which demonstrate REM sleep without atonia (RWA). Finally, the disturbance is not better explained by another sleep disorder, mental disorder, medication or substance abuse^18^. RBD is another non-motor symptom frequently observed in all stages of PD, with a reported pooled prevalence of 42.3%^19^. One of the clinical manifestations observed in early PD, RBD is strongly associated with more severe motor and non-motor symptoms, including cognitive impairment^20–22^. Several recent studies have indicated that RBD represents an early phase of α-synucleinopathy^23^. In fact, those with idiopathic RBD have a high risk of developing PD, dementia with Lewy bodies (DLB) and multiple system atrophy (MSA)^24^. The concept of sleep disorder as a prodromal phase of α-synucleinopathy has attracted growing interest as early intervention and treatment of RBD could have a neuroprotective effect and subsequently modify disease progression^25,26^.

Mild cognitive impairment (MCI) is among the most frequently reported non-motor symptoms in PD, with a prevalence of approximately 30%^27^. Characteristic cognitive phenotypes observed in early PD include mild deficits in attention, executive function, verbal memory, and visuospatial domains, involving dopaminergic frontostriatal as well as non-dopaminergic posterior temporal and parietal regions of the brain^28^. In the early stages of PD, subtle cognitive deficits, such as bradyphrenia, mild aphasia and planning initiation difficulty are frequently observed in PD patients^29^. Cognitive impairment in PD is of paramount importance as it negatively affects the patient’s quality of life and independence, resulting in a great burden for caregivers, and tremendous economic costs^30,31^.

Identification of factors that may contribute to cognitive dysfunction is imperative given that PD patients have a sixfold higher risk for developing dementia compared to the general population^32^. Both constipation and RBD are identified as early signs of PD which may be associated with cognitive impairment among PD patients. Most importantly, RBD and constipation appear to be the earliest non-motor symptoms that can occur during the prodromal phase of PD^33,34^. However, whether constipation is linked to subsequent cognitive impairment in the course of PD remains unclear, and the possibility that constipation plays a synergistic role with RBD in cognitive performance is uncertain. By accessing the Parkinson’s Progression Markers Initiative (PPMI) database, we aimed to answer the above questions in this study.

## Methods

### Dataset and study participants

PPMI is an ongoing, prospective cohort study launched in 2010 funded by the Michael J. Fox Foundation to comprehensively assess the progression of clinical symptoms, advanced imaging and biologic sampling in newly diagnosed PD patients with 5 years follow-up. The aim, methodology, and study assessments have been reported elsewhere and are available on the PPMI website. Permission was granted to access the PPMI data. To our knowledge, PPMI is the largest comprehensive study of PD patients with a longitudinal design carried out to date, that is freely available.

Enrolled participants were over 30 years old, with a recent PD diagnosis. The inclusion criteria required that PD subjects had (1) at least two of the following clinical phenotypes: resting tremor, bradykinesia or rigidity (must have either resting tremor or bradykinesia), (2) a PD diagnosis made within 2 years of baseline assessment, (3) Hoehn and Yahr stage I or II at recruitment, and (4) dopamine transporter deficit on single-photon emission computed tomography (SPECT) scan. Subjects were assessed at baseline and followed for 5 years, over which some subjects commenced dopamine replacement therapy (DRT) such as levodopa, dopamine agonist and others.

The overall PPMI study was approved by the Research Subjects Review Board at the University of Rochester, and the study obtained institutional approval from all 33 participating sites in United States, Europe, Israel and Australia. Written informed consent was acquired from each participant before being included in the study. PPMI study was performed in accordance with the relevant guidelines and regulations.

### Demographic characteristics

Disease duration at enrolment was calculated in months from the date of PD diagnosis. Body-mass-index (BMI) was calculated in kg/m^2^ using participants’ height in metres squared (m^2^) and weight in kilograms (kg).

### Dopamine replacement therapy (DRT)

The dose of DRT prescription at year 5 follow-up visit was expressed in levodopa equivalent daily dose (LEDD) in milligrams (mg).

### Constipation

Constipation was classified as occurring at least sometimes when measured using the Scale for Outcomes in PD for Autonomic Symptoms (SCOPA-AUT) questionnaire^35^. The specific questions relating to gastrointestinal function utilised in the current analysis were:

1. Constipation is a blockage of the bowel, a condition in which someone has a bowel movement twice a week or less. In the past month, have you had problems with constipation?
2. In the past months, did you have to strain hard to pass stools? In the past month, have you had involuntary loss of stools?

For each question, response options included were “never”, “sometimes”, “regularly”, and “often”. For the purpose of this evaluation, gut symptoms were defined as occurring “never”, “sometimes”, regularly” or “often”, with scores of 0, 1, 2, 3 respectively allocated. The score for constipation for each participant will be the sum of scores for questions (1) and (2).

### REM sleep behaviour disorder

For the diagnosis of probable REM sleep behaviour disorder (pRBD), the REM Sleep Behaviour Disorders Screening Questionnaire (RBDSQ) was used^36^. An RBDSQ score of 6 and above was considered as a positive test result. A cut-off score of 6 was reported by Chahine et al. as a strong test performance with a sensitivity and specificity of 74.2% and 79.4% respectively^37,38^.

### Neuropsychological tests

At baseline and 5 years following PD diagnosis, the following cognitive tests were administered as measures of the cognitive domain indicated:

1. Montreal Cognitive Assessment (MoCA): global cognitive function^39^
2. Symbol Digit Modalities Test (SDMT): processing speed/attention^40^
3. Letter Number Sequencing (LNS): executive function/working memory
4. Semantic fluency: executive function/working memory^41^
5. Benton Judgment-of-Line-Orientation (JLO) 15-item (split-half) version: visuospatial function^42^
6. Hopkins Verbal Learning Test-Revised (HVLT-R) immediate, delayed recall discrimination recognition and retention: verbal learning and memory^43^

### Statistical methods

Statistical analyses were run using the Statistical Package for the Social Sciences (SPSS) statistics (version 25, SPSS, Inc., Chicago, IL). Statistical significance was set at *p* ≤ 0.01. Assumptions for statistical tests were checked and fulfilled if required. In order to incorporate missing data, the feature of approximate Bayesian bootstrap is attempted in SPSS to confirm the results^44^. Regarding multiple testing, we used Bonferroni correction to examine differences among different groups.

Baseline demographic characteristics of the PPMI participants were summarised using descriptive statistics. Descriptive statistics are reported as means and standard deviation (SD) with range. One-way analysis of variance (ANOVA) was applied to examine the data of male and female PD subjects at baseline and year 5 follow-up visit.

Spearman’s rank order correlation analysis was conducted to investigate the effects age, BMI and years of education on constipation score and neuropsychological test performances. Those variables of interest reaching a significance threshold of *p* ≤ 0.01 underwent further linear mixed-effects modelling as covariates.

Furthermore, linear mixed-effects modelling was run to evaluate longitudinal data to account for multiple testing. The independent and combined effects of baseline constipation and pRBD on cognitive outcomes in the PD cohort were assessed for each neuropsychological measure. The interaction effect of constipation and sleep disorder on cognitive outcomes of interest was analysed based on the following responses: (1) presence of probable RBD (pRBD) when RBDSQ score was ≥ 6 and (2) presence of constipation when constipation score was > 3. In each model, the follow-up year, presence or absence of pRBD and constipation at baseline were the independent variables. In order to examine the synergistic effect of baseline constipation and pRBD on cognitive outcomes, the independent variable was the constipation*pRBD interaction term. Age, years of education, gender and LEDD were treated as fixed effects in the models. The dependent variables were the repeated scores of each neuropsychological measure over 5 years. Patient identifier was incorporated as random effect in the analysis to allow for interindividual variability for baseline neuropsychological test scores. Other random effect included was the constipation*pRBD*follow-up year.

The discrimination ability of both constipation and pRBD was then assessed by area under the curve of a receiver operating characteristic (ROC) using different cut-off scores for SDMT. SDMT cut-off scores of 28 and 34 were adopted based on previous studies to capture 15% of rapid PD progressors^45^ and 28% PD subjects with worse SDMT scores, which was equivalent to a MoCA cut-off score of 26 in this study.

## Results

### Participant characteristics

The sample for this study included newly diagnosed, untreated PD patients (n = 360), who were enrolled into the PPMI study between June, 2010 and April, 2013 with 5 years follow-up assessments. Baseline characteristics of PD subjects are shown in Table 1, with a comparison of clinical variables in men and women. Participants who did not have both baseline constipation, sleep disorders and neuropsychological data were excluded. The PD cohort comprised of 238 male participants and 122 female participants. The male and female PD subjects did not differ in demographic characteristics, apart from male subjects having a greater BMI (27.82 ± 4.55) compared to the female subjects (25.45 ± 4.62) (*p* < 0.001, Table 1). At baseline, male subjects had a significantly lower MoCA score (males vs females: 26.94 ± 2.05 vs 27.60 ± 2.28, *p* = 0.008), SDMT score (males vs females: 40.32 ± 9.77 vs 44.36 ± 9.35, *p* < 0.001), HVLT discrimination recognition score (males vs females: 10.07 ± 1.40 vs 10.76 ± 2.17, *p* < 0.001), HVLT-R immediate recall score (males vs females: 23.73 ± 4.90 vs 26.36 ± 4.60, *p* < 0.001) and HVLT-R delayed recall score (males vs females: 8.03 ± 2.56 vs 9.35 ± 2.09, *p* < 0.001) (Table 1).

**Table 1:** Characteristics of PPMI subjects with PD diagnosis at recruitment.

	PD subjects (n = 360)	Male subjects (n = 238)	Female subjects (n = 122)	*p*
Baseline demographic characteristics				
Age, years	61.23 (9.75) (33.5, 84.9)	61.77 (9.99) (34.8, 84.9)	60.15 (9.22) (33.5, 81.8)	0.14
No. of subjects aged > 65	141 (39.2%)	102 (42.9%)	39 (32%)	n/a
BMI	27.01 (4.62) (16.7, 43.8)	27.82 (4.55) (19.8, 41.6)	25.45 (4.62) (16.7. 43.8)	** < 0.001**
Duration since clinical diagnosis, months	6.39 (6.36) (0, 36)	6.13 (5.77) (0, 35)	6.91 (7.44) (1, 36)	0.29
Education, years	15.57 (2.94) (5, 26)	15.72 (3.01) (5, 26)	15.27 (2.85) (5, 22)	0.17
Dopaminergic replacement therapy				
No of subjects on DRT at year 5	329 (91.4%)	223 (93.7%)	106 (86.9%)	n/a
LEDD	581.6 (712.4) (507, 655)	581.2 (721.6) (4687, 675)	582.2 (700.7) (461, 704)	0.99
Gastrointestinal symptoms				
Constipation score	1.00 (1.25) (0, 6)	0.96 (1.22) (0, 6)	1.08 (1.30) (0, 6)	0.37
No. of subjects with constipation score > 3	17 (4.7%)	10 (4.2%)	7 (5.7%)	n/a
Sleep behavior disorders				
RDBSQ	3.99 (2.64) (0, 12)	4.16 (2.79) (0, 12)	3.66 (2.30) (0, 12)	0.09
No. of subjects with RBDSQ > 5	87 (24.2%)	48 (20.2%)	39 (32%)	n/a
Neuropsychological tests				
MoCA	27.16 (2.22) (19, 30)	26.94 (2.05) (19, 30)	27.60 (2.28) (21, 30)	**0.008**
No. of subjects with MoCA < 26	80 (22.2%)	55 (23.1%)	25 (20.5%)	n/a
SDMT	41.68 (9.81) (7, 82)	40.32 (9.77) (7, 76)	44.36 (9.35) (7, 82)	** < 0.001**
LNS	10.68 (2.60) (4, 20)	10.54 (2.70) (4, 20)	10.95 (2.38) (5, 20)	0.16
Semantic fluency	49.42 (11.73) (20, 103)	49.67 (12.28) (20, 103)	48.94 (10.61) (25, 81)	0.58
JLO	13.00 (1.96) (4, 15)	13.01 (1.90) (7, 15)	12.98 (2.08) (4, 15)	0.87
HVLT-R discrimination recognition	10.30 (1.70) (0, 17)	10.07 (1.40) (0, 17)	10.76 (2.17) (7, 15)	** < 0.001**
HVLT-R immediate recall	24.62 (4.95) (9, 36)	23.73 (4.90) (9, 35)	26.36 (4.60) (13, 36)	** < 0.001**
HVLT-R delayed recall	8.47 (2.49) (0, 12)	8.03 (2.56) (0, 12)	9.35 (2.09) (4, 12)	** < 0.001**
HVLT-R retention	1.08 (0.23) (0, 2.43)	1.09 (0.25) (0, 2.43)	1.06 (0.19) (0.64, 1.75)	0.23

Data are shown as mean (SD) (range) or frequency (%).

Data with disease duration were missing for 8 male subjects and 8 female subjects.

All the data between male and female PD subjects were analysed using one-way ANOVA.

BMI, Body Mass Index; HVLT-R, Hopkins Verbal Learning Test-Revised; JLO, Benton Judgement of Line Orientation; LEDD, Levodopa equivalent daily dose; LNS, Letter Number Sequencing; MoCA, Montreal Cognitive Assessment; RBDSQ, Rapid eye movement sleep behaviour disorder questionnaire; SDMT, Symbol Digit Modalities Test; n/a, Not applicable.

At year 5 follow-up, data were available on 281 PD subjects with 189 male and 92 female participants (Supplementary Table S1). Female subjects had better scores on several neuropsychological tests, such as MoCA score (males vs females: 26.24 ± 3.77 vs 27.38 ± 2.86, *p* = 0.01), SDMT score (males vs females: 37.80 ± 12.71 vs 43.54 ± 12.42, *p* < 0.001), LNS (males vs females: 9.74 ± 3.14 vs 10.67 ± 2.65, p = 0.01) and semantic fluency score (males vs females: 45.67 ± 12.56 vs 55.42 ± 12.37, *p* < 0.001) (Supplementary Table S1).

### Effect of constipation or pRBD on cognitive outcomes in PD

At baseline, constipation, MoCA, SDMT, LNS, HVLT-R discrimination recognition, HVLT-R immediate recall and HVLT-R delayed recall were associated with age. Years of education was positively associated with baseline SDMT score (*r*_*s*_ = 0.140, *p* = 0.008), HVLT discrimination recognition score (*r*_*s*_ = 0.147, *p* = 0.005), HVLT-R immediate recall score (*r*_*s*_ = 0.173, *p* = 0.001) and HVLT-R delayed recall score (*r*_*s*_ = 0.193, *p* < 0.001)*.* No associations were identified between baseline constipation score or cognitive outcomes and BMI at baseline observation (Supplementary Table S2).

In the linear mixed-effect model, in males, RBDSQ score at baseline was associated with SDMT (*p* = 0.009) over 5 years (Table 2). No associations were observed between either constipation or RBDSQ with the neuropsychological tasks, MoCA, LNS, JLO, HVLT-R and semantic fluency in both genders (Table 2).

**Table 2:** The effects of baseline constipation and RBDSQ on cognitive outcomes over 5 years.

Clinical scores at baseline	Neuropsychological tests																	
	MoCA ^1^		SDMT ^2^		LNS ^2^		Semantic fluency ^1^		JLO ^1^		HVLT-R discrimination recognition ^2^		HVLT-R immediate recall ^2^		HVLT-R delayed recall ^2^		HVLT-R retention ^1^	
	β	*p*	β	*p*	β	*p*	β	*p*	β	*p*	β	*p*	β	*p*	β	*p*	β	*p*
Constipation score																		
Overall	− 0.76 (− 1.54, 0.03)	0.06	0.69 (− 1.82, 3.2)	0.59	0.09 (− 0.59, 0.77)	0.79	− 1.63 (− 4.64, 1.39)	0.29	− 0.15 (− 0.81, 0.51)	0.65	− 0.02 (− 0.68, 0.63)	0.95	0.84 (− 0.85, 2.52)	0.32	0.1 (− 0.72, 0.92)	0.80	− 0.04 (− 0.17, 0.1)	0.57
Male	− 0.74 (− 1.75, 0.27)	0.15	4.3 (0.6, 7.99)	0.02	0.94 (− 0.04, 1.92)	0.06	− 4.06 (− 8.53, 0.41)	0.08	− 0.61 (− 1.51, 0.30)	0.19	− 0.71 (− 1.48, 0.05)	0.07	0.09 (− 1.97, 2.15)	0.93	− 0.56 (− 1.58, 0.46)	0.28	− 0.1 (− 0.25, 0.05)	0.20
Female	− 0.7 (− 1.72, 0.31)	0.17	− 1.17 (− 4.48, 2.15)	0.49	− 0.34 (− 1.27, 0.59)	0.47	0.48 (− 3.73. 4.69)	0.82	0.22 (− 0.58, 1.03)	0.59	0.69 (− 0.13, 1.52)	0.10	2.39 (0.32, 4.47)	0.02	1.06 (0.08, 2.04)	0.03	0.005 (− 0.14, 0.15)	0.95
RBDSQ																		
Overall	− 1.01 (− 1.93, 0.1)	0.03	0.88 (− 2.19, 3.95)	0.58	0.83 (− 1.66, − 0.01)	0.05	− 0.53 (− 4.22, 3.16)	0.78	− 0.4 (− 1.16, 0.37)	0.31	− 0.19 (− 0.92, 0.53)	0.60	0.79 (− 1.13, 2.71)	0.42	0.42 (− 0.53, 1.37)	0.38	− 0.09 (− 0.23, 0.05)	0.23
Male	− 0.97 (− 2.13, 0.17)	0.09	5.59 (1.38, 9.81)	**0.009**	− 0.08 (− 1.19, 1.04)	0.89	− 0.87 (− 5.95, 4.21)	0.74	− 0.88 (− 1.88, 0.12)	0.09	− 0.7 (− 1.55, 0.05)	0.10	1.49 (− 0.82, 3.8)	0.21	0.39 (− 0.77, 1.54)	0.51	− 0.19 (− 0.35, − 0.03	0.02
Female	− 1.11 (− 2.48, 0.27)	0.11	− 4.2 (− 8.78, 0.37)	0.07	− 1.39 (− 2.68, − 0.11)	0.03	− 2.82 (− 8.62, 2.98)	0.34	0.07 (− 1.04, 1.17)	0.90	0.17 (− 0.83, 1.17)	0.74	− 0.43 (− 3.18, 2.33)	0.76	0.15 (− 1.2, 1.49)	0.83	0.05 (− 0.14, 0.25)	0.59
Constipation*RBDSQ																		
Overall	− 0.02 (− 1.07, 1.02)	0.96	− 5.55 (− 8.8, − 2.3)	**0.001**	− 0.002 (− 0.88, 0.88)	1.00	− 1.42 (− 5.33, 2.5)	0.48	− 0.13 (− 1.01, 0.74)	0.76	− 0.12 (− 1.01, 0.78)	0.80	− 2.36 (− 4.63, − 0.1)	0.04	− 0.98 (− 2.08, 0.11)	0.08	0.13 (− 0.05, 0.31)	0.15
Male	− 0.28 (− 1.49, 0.94)	0.66	− 9.53 (− 13.98, − 5.08)	** < 0.001**	− 0.91 (− 2.1, 0.28)	0.13	− 1.19 (− 6.61, 4.24)	0.67	0.26 (− 0.87, 1.4)	0.65	0.56 (− 0.4, 1.52)	0.25	− 2.38 (− 4.93, 0.17)	0.07	− 0.67 (− 1.92, 0.58)	0.29	0.25 (0.05, 0.44)	0.02
Female	0.34 (− 1.15, 1.83)	0.65	− 1.23 (− 6.09, 3.64)	0.62	0.84 (− 0.53, 2.2)	0.23	1.1 (− 5.07, 7.25)	0.73	− 0.49 (− 1.66, 0.69)	0.42	− 0.7 (− 1.9, 0.49)	0.24	− 2.00 (− 5.02, 1.03)	0.19	− 1.09 (− 2.53, 0.34)	0.14	− 0.02 (− 0.24, 0.19)	0.83

The effects of constipation and RBDSQ on cognitive outcomes over 5 years in PD subjects were analysed using linear mixed-effects modelling adjusted for 1: age and LEDD; 2: age, years of education and LEDD based on gender and adjusted for gender when analysing the overall cohort. In each model, presence/absence of constipation, RBDSQ, constipation*RBDSQ interactive term are the independent variables whereas the neuropsychological test scores are the dependent variables.

β, β-coefficient (95% CI); HVLT-R, Hopkins Verbal Learning Test-Revised; JLO, Benton Judgement of Line Orientation; LEDD, Levodopa equivalent daily dose; LNS, Letter Number Sequencing; MoCA, Montreal Cognitive Assessment; RBDSQ, Rapid eye movement sleep behaviour disorder questionnaire; SDMT, Symbol Digit Modalities Test.

### Synergistic effect of the interaction between constipation and RBD on cognitive outcomes in PD

When constipation and RBD scores were dichotomised into those with or without those conditions, a significant interaction between the presence of constipation and pRBD at baseline was present in their association with SDMT (*p* < 0.001) in male subjects only (Table 2). The SDMT score amongst male PD subjects with both constipation and pRBD at baseline declined by 9.53 points more than in those without both constipation and pRBD on follow-up.

While excluding PD subjects with MoCA < 26, three neuropsychological tests, namely SDMT (*p* < 0.001), LNS (*p* = 0.003) and HVLT-R retention (*p* = 0.01) appeared to be significant in their interaction with both constipation and pRBD in male subjects (Supplementary Table S3). In females, presence of pRBD was associated with MoCA (*p* = 0.005) and semantic fluency (*p* = 0.005) scores respectively whereas constipation was associated with MoCA score (*p* = 0.003) only.

When a cut-off score of 28 for SDMT was applied, the prediction accuracy for cognitive impairment over 5 years for all parameters (constipation, pRBD, age, years of education and LEDD) (Supplementary Fig. S1A) was higher than when a SDMT cut-off of 34 was applied in male subjects (Supplementary Fig. S1B). Combining constipation and pRBD with other demographic variables (age, years of education and LEDD) resulted in a higher AUC compared with constipation or pRBD alone when a SDMT cut-off of 28 was applied (AUC 0.79 [95% CI 0.694–0.892, *p* < 0.001] vs constipation alone: AUC 0.63 [95% CI 0.586–0.687, *p* < 0.001] vs pRBD alone: AUC 0.61 [95% CI 0.561–0.662, *p* < 0.001]) (Supplementary Fig. S1A).

## Discussion

### Role of gender in cognitive outcomes

In this study of an early drug-naïve PD cohort, we observed significant gender differences in performance on cognitive tasks at the early stages of PD. At enrolment, men performed significantly worse than women on cognitive tasks that measure global cognition, speed-attention processing, verbal learning and memory.

Previous studies of PD have documented certain PD clinical characteristics that are more frequently seen in men than women, including cognitive dysfunction and rigidity, while dyskinesias and depression are more commonly observed in women^46,47^. The male preponderance in PD could be due to the absence of the neuroprotective role of oestrogen in men. Haaxma and coworkers^48^ proposed a protective role of oestrogen as hyperestrogenic factors such as parity, later age for menopause, and duration of reproductive life are associated with later age at onset of PD in women^48^. The possibility of sex steroid hormones underpinning these gender differences is demonstrated in animal and clinical studies. A study in primates showed that oestrogen regulates the production and metabolism of dopamine while modulating the expression and function of the dopamine receptor^49^. The protective role of oestrogen in women is underlined by the observation that postmenopausal oestrogen therapy was found to be associated with a lower risk of PD in women^50^. These data clearly suggest a key role of oestrogen in delaying loss of dopaminergic neurons while preserving a functional nigrostriatal dopaminergic pathway^49^. In conjunction with these observations, our findings suggest that cognition in men deteriorates more rapidly and that the impact of PD on men is more prominent than in women.

Regardless of the large body of research findings, the neuroprotective effect of oestrogen on the nigrostriatal dopaminergic pathway alone is not adequate to explain a more severe clinical phenotype in men with PD. Not all studies investigating the role of oestrogen in PD have found beneficial effect of oestrogen, such as a large prospective cohort study in Denmark, which comprised 27,466 women shows that oestrogen does not alter the risk of PD in women^51^. The conflicting findings suggest that other factors, such as genetics and environmental factors may also be responsible for the gender differences reported in this study^52^. We also note that there were great numbers of male than female patients in this data set, so it is possible that statistical power may have influenced the findings to some degree.

### Influence of pRBD on cognitive outcomes

Our study has shown that baseline RBDSQ score was strongly associated with measures of processing speed and attention in male subjects. In agreement with our findings, a study based on the Oxford Parkinson’s disease center (OPDC) cohort found that pRBD was significantly associated with cognitive impairment (*p* = 0.006)^53^. Previous studies have indicated pRBD as a risk factor for cognitive impairment and deterioration in advanced PD^54–56^. However, more recent studies have shown that pRBD is associated with worse motor and cognitive performances, indicating a more extensive spread of α-synucleinopathy in patients diagnosed with pRBD^57,58^.

Previous work on the PPMI dataset up to the year 3 follow-up visit demonstrated pRBD at baseline was associated with a greater rate of cognitive decline, specifically in attention and memory domains^38^. The present study, investigating this association over a 5-year period following PD diagnosis reports similar findings. Interestingly, our further analysis revealed that this association was only present in men—an important finding that was not reported previously, which suggests there may be a gender difference in progression of cognitive decline.

### Synergistic effect of the interaction between constipation and RBD on cognitive outcomes

Our study revealed that constipation and pRBD at baseline, in combination, had a significant effect on cognitive performance after 5 years following diagnosis of PD. We observed a synergistic effect of the interaction between constipation and pRBD at baseline on measures of processing speed/attention, verbal learning and memory in men. However, the synergistic effect of constipation and pRBD did not impact any neuropsychological test measure in women. These observations indicate the synergistic effect of constipation and pRBD on cognitive performance was more prominent in men than in women. The synergistic effect of constipation and pRBD produced good discrimination for the prediction of cognitive impairment, particularly measure of processing speed/attention while the addition of demographic variables such as age, years of education and LEDD further improved the predictive value. Demographic variables such as age and educational level are known to be associated with cognitive decline in the PD cohort as demonstrated in a cross-sectional study that utilized the Parkinson’s disease cognitive study (PACOS) data^27^. Further validation of this finding is warranted to determine whether constipation and pRBD scores can be utilised to predict future attention, motor speed, verbal learning and memory performance in men with PD.

Our results reported that pRBD, independently and in combination with constipation, was related with the cognitive domains, namely processing speed and attention in men, suggesting that pRBD, constipation and cognitive impairment affecting processing speed and attention domains may be attributed to a similar neurodegenerative process, particularly in men. In contrast, LNS, JLO and semantic fluency, which are measures of visuospatial function, executive function and working memory did not seem to relate to either baseline constipation or pRBD in both genders. Our result shows that these specific cognitive domains are less likely to be impacted as PD progresses.

On the other hand, the concept of the gut-brain axis has long been investigated to describe the connection between the gastrointestinal tract and the CNS for a better understanding of the pathogenesis of PD^59–61^. A dual-hit hypothesis was postulated by Braak and colleagues, whereby evaluation of 413 brain autopsies led Braak and colleagues to propose that a neurotropic pathogen initiates PD pathology in the gut, which then spreads to the brain via both nasal and gastric routes^62^. The gut-originated pathogen appears to propagate to the cortical regions of the brain to cause cognitive dysfunction in PD^63^. Pan-Montojo and coworkers successfully induced PD-like pathology in mice by oral administration of rotenone, a mitochondrial complex I inhibitor in their Dresden Parkinson Model^64^. In addition, post-mortem observations of PD patients suggest that the degenerative process in PD may begin in the peripheral autonomic nervous system before affecting the CNS^65^. Our speculation that there might be a connection between gastrointestinal dysfunction and cognitive impairment in PD is supported by the association between baseline constipation and RBD scores with cognitive performance on follow-up, particularly in men.

In short, the non-motor symptoms of PD, specifically constipation and REM sleep disorder have an association with future cognitive impairment in PD, where gender may play a role in modifying this association. The findings of this study require further validation and extension by screening a larger cohort to allow assessment of the utility of constipation and sleep disorder scores as a tool to predict future cognitive impairment in PD.

### Limitations and study improvement

There are a number of limitations in this study. Most importantly, the PPMI cohort generally comprised highly educated and Caucasian volunteers, which reduces the generalisability of the study outcomes. While there are multiple approaches to measure the episodes and severity of constipation, SCOPA-AUT relies on self-reported constipation. Sufferers’ definition and interpretation of constipation vary widely, which makes constipation a subjective experience, thus it can be difficult to understand what symptoms are considered as “real” constipation^66^. The answer choices for SCOPA-AUT: “never”, “sometimes”, “regularly” and “often” are also subjective to the participants and may not accurately represent the frequency of episodes or severity of constipation. Another study that utilised a variety of questionnaires to evaluate self-reported constipation found that generally PD subjects suffered objective colonic dysfunction more frequently than they reported constipation^67^. Furthermore, according to the International Classification of Sleep Disorders (ICSD), the gold standard for measuring sleep disorders is polysomnography. However, there was no access to polysomnography data in the PPMI cohort. Therefore, our study might require better measures to quantify or define constipation and pRBD for the administration of self-assessment questionnaires such as SCOPA-AUT and RBDSQ.

Furthermore, studies have indicated there is a possible practice effect from previous exposure to a neuropsychological assessment, resulting in an increasing familiarity on the administration of the neuropsychological assessment^68,69^. Repeated administration of the same neuropsychological assessments in this study might result in an improvement on scores across the 5-year period, which subsequently reduce the accuracy and reliability of our analyses. This emphasises the need to take into consideration the practice effect that might arise when interpreting cognitive changes across time.

The statistical method, linear mixed-effect modelling, does not account for the varying sample size of the PD cohort at different annual follow-up visits. Attrition during follow-up is often inevitable and could lead to a biased result^70^. Despite the lower number of PD participants at follow-up visits, the correlation between baseline constipation, pRBD and cognitive performance over 5 years period remained significant. In order to better comprehend the true effects of baseline constipation and pRBD on future cognitive outcomes, this study has included sub-analysis, such as gender-stratified model. The cognitive outcomes were measured by nine neuropsychological scores and they represent a set of inferences simultaneously, which are likely to incur erroneous inferences. Thus, stringent significance threshold, p value no more than 0.01 in this study*,* is required to compensate for the possible uncertainty in results due to multiple comparisons.

## Conclusion

Despite the limitations of this study, our analysis showed that constipation and pRBD are strongly associated with worse cognitive outcomes in PD, while specific cognitive features appear to be more affected in males. Therefore, future work should address whether screening for constipation and sleep disorder might assist with early detection of cognitive impairment in men, to allow a more thorough understanding of the progression and prognosis of cognitive impairment among this sub-group of PD patients.

## Ethics approval and consent to participate

Permission and approval for conducting this study was received from the Human Research Advisory Panel (HREAP) Executive of UNSW Sydney, under approval number of HC180099.

## Supplementary information

Supplementary information.

## Data Availability

Data used in the preparation of this article were obtained from the Parkinson’s Progression Markers Initiative (PPMI) database (www.ppmi-info.org/data). For up-to-date information on the database, visit www.ppmi-info.org. All data generated or analysed during this study are included in this published article and its supplementary information files.
