# Supplementary material for: Constipation and sleep behaviour disorder associate with processing speed and attention in males with Parkinson’s disease over five years follow-up
Source: Sci Rep. 2020 Nov 4;10:19014. doi: 10.1038/s41598-020-75800-4 (PMC7643116; doi:10.1038/s41598-020-75800-4)
Supplement: Supplementary file 1 — Supplementary information. [file 41598_2020_75800_MOESM1_ESM.pdf]

Constipation and Sleep Behaviour Disorder Associate with Processing Speed and Attention in Males with Parkinson's Disease over Five Years Follow-up

Wee Lee Kong[1], Yue Huang[1,2]*, Elizabeth Qian[1], Margaret J Morris[1]*

1. Department of Pharmacology, School of Medical Sciences, Faculty of Medicine, UNSW Sydney, 2052, Australia
2. China Clinical Research Centre for Neurological Diseases, Beijing Tiantan Hospital, Capital Medical University, 100071, China

Correspondence to: Prof. Margaret Morris, email: m.morris@unsw.edu.au; Prof. Yue Huang, email: yue.huang@ncrcnd.org.cn

Email addresses: Wee Lee Kong (z5028557@unsw.edu.au); Elizabeth Qian (z3414477@unsw.edu.au)

Supplementary Table S1. **Constipation and cognitive scores in the PD cohort at year 5 follow-up.**

**Variables**	**Assessment visit at year 5**			
	**PD subjects**	**Male**	**Female**	***p***
**No. of PD subjects**	281	189	92	
**Gastrointestinal symptoms**				
Constipation	1.82 (1.65) (0, 7)	1.79 (1.63) (0,7)	1.86 (1.77) (0,6)	0.76
**Sleep behavior disorders**				
RBDSQ	4.91 (3.21) (0, 13)	5.23 (3.26) (0, 13)	4.25 (3.01) (0, 12)	0.02
**Neuropsychological tests**				
MoCA	26.61 (3.53) (2, 31)	26.24 (3.77) (2, 31)	27.38 (2.86) (15, 30)	**0.01**
No. of subjects with MoCA<26	93 (33.1%)	53 (28%)	40 (43.5%)	n/a
SDMT	39.68 (12.88) (0, 74)	37.80 (12.71) (0, 69)	43.54 (12.42) (1, 74)	**<0.001**
LNS	10.04 (3.02) (1, 20)	9.74 (3.14) (1, 20)	10.67 (2.65) (2, 18)	**0.01**
Semantic fluency	48.86 (13.29) (7, 90)	45.67 (12.56) (7, 90)	55.42 (12.37) (11, 88)	**<0.001**
JLO	12.44 (2.28) (4, 15)	12.69 (2.23) (4, 15)	11.95 (2.32) (5, 15)	**0.01**
HVLT-R discrimination recognition	11.10 (1.36) (4, 14)	11.08 (1.37) (4, 13)	11.15 (1.34) (6, 14)	0.67
HVLT-R immediate recall	25.89 (6.34) (4, 36)	25.75 (6.43) (4, 36)	26.17 (6.17) (12, 36)	0.60
HVLT-R delayed recall	9.12 (3.04) (0, 12)	9.01 (3.14) (0, 12)	9.35 (2.83) (0, 12)	0.38
HVLT-R retention	1.16 (0.42) (0.5, 6.5)	1.17 (0.48) (1, 7)	1.13 (0.28) (1, 3)	0.39

Data are shown as mean (SD) (range).
All the data between male and female PD subjects were analyzed using one-way ANOVA.

Abbreviations

HVLT-R: Hopkins Verbal Learning Test-Revised; JLO: Benton Judgement of Line Orientation; LNS: Letter Number Sequencing; MoCA: Montreal Cognitive Assessment; RBDSQ: Rapid eye movement sleep behaviour disorder questionnaire; SDMT: Symbol Digit Modalities Test

Supplemetary Table S2. **Spearman rank-order correlation analysis between demographic variables and clinical variables at baseline**

**Demographic variables**		**Clinical variables**				**Neuropsychological tests**																	
		**Constipation score**		**RBDSQ**		**MoCA**		**SDMT**		**LNS**		**Semantic fluency**		**JLO**		**HVLT-R discrimination recognition**		**HVLT-R immediate recall**		**HVLT-R delayed recall**		**HVLT-R retention**	
		r_s	p	r_s	p	r_s	p	r_s	p	r_s	p	r_s	p	r_s	p	r_s	p	r_s	p	r_s	p	r_s	p
**Age**	Overall	0.239	<**0.001**	-0.007	0.90	-0.218	<**0.001**	-0.514	<**0.001**	-0.373	<**0.001**	-0.063	0.23	0.087	0.10	-0.217	<**0.001**	-0.279	<**0.001**	-0.304	<**0.001**	0.032	0.54
	Male	0.307	<**0.001**	0.076	0.24	-0.259	<**0.001**	0.476	<**0.001**	-0.395	<**0.001**	-0.049	0.45	0.098	0.13	-0.191	**0.003**	-0.252	<**0.001**	-0.296	<**0.001**	0.038	0.55
	Female	0.110	0.23	-0.220	0.02	-0.105	0.25	-0.537	<**0.001**	-0.302	**0.001**	-0.107	0.24	0.050	0.59	0.222	**0.01**	-0.295	**0.001**	-0.296	**0.001**	0.019	0.84
**BMI**	Overall	-0.098	0.06	0.029	0.59	-0.046	0.39	0.012	0.82	0.019	0.72	0.026	0.63	-0.041	0.43	-0.108	0.04	-0.131	**0.01**	-0.120	0.02	0.022	0.67
	Male	-0.110	0.09	-0.059	0.37	-0.047	0.47	0.038	0.56	-0.014	0.83	0.006	0.93	-0.084	0.20	-0.088	0.18	-0.103	0.11	-0.130	0.05	0.011	0.86
	Female	-0.045	0.63	0.157	0.08	-0.043	0.64	0.116	0.21	0.119	0.20	0.059	0.52	0.010	0.91	-0.018	0.85	0.036	0.69	0.095	0.30	0.010	0.91
**Years of education**	Overall	-0.080	0.13	0.013	0.80	0.059	0.27	0.140	**0.008**	0.127	0.02	0.041	0.44	0.044	0.40	0.147	**0.005**	0.173	**0.001**	0.193	<**0.001**	-0.081	0.13
	Male	-0.114	0.08	0.040	0.55	0.046	0.48	0.119	0.07	0.132	0.04	0.012	0.86	0.069	0.29	0.131	0.04	0.174	**0.007**	0.192	0.003	-0.083	0.20
	Female	-0.004	0.97	-0.064	0.48	0.112	0.22	0.250	**0.006**	0.136	0.14	0.100	0.27	-0.005	0.95	0.227	0.01	0.249	**0.006**	0.276	**0.002**	-0.111	0.23

The associations between demographic variables and clinical variables at baseline were analyzed using Spearman rank-order correlation analysis.

Abbreviations

BMI: Body Mass Index; female ref: female gender was set as the reference; HVLT-R: Hopkins Verbal Learning Test-Revised; JLO: Benton Judgement of Line Orientation; LNS: Letter Number Sequencing; MoCA: Montreal Cognitive Assessment; RBDSQ: Rapid eye movement sleep behavior disorder questionnaire; SDMT: Symbol Digit Modalities Test; r_s: Spearman correlation coefficient

Supplementary Table S3. **The effects of baseline constipation and RBDSQ on cognitive outcomes over 5 years while excluding PD subjects with MoCA<26**

**Clinical scores at baseline**		**Neuropsychological tests**																	
		**MoCA** [1]		**SDMT** [2]		**LNS** [2]		**Semantic fluency** [1]		**JLO** [1]		**HVLT-R discrimination recognition** [2]		**HVLT-R immediate recall** [2]		**HVLT-R delayed recall** [2]		**HVLT-R retention** [1]	
		**β**	***p***	**β**	***p***	**β**	***p***	**β**	***P***	**β**	***p***	**β**	***p***	**β**	***p***	**β**	***p***	**β**	***p***
**Constipation score**	Overall	-1.18(-2.26, 0.1)	0.03	0.98(-1.85, 3.82)	0.45	0.18(-0.57, 0.94)	0.64	-2.79(-6.4, 0.82)	0.13	-0.22(-0.88, 0.44)	0.51	-0.03(-0.66, 0.61)	0.94	0.73(-0.89, 2.35)	0.37	0.15(-0.63, 0.92)	0.71	-0.03(-0.18, 0.12)	0.69
	Male	0.08(-1.13, 1.29)	0.89	7.22(2.59, 11.86)	**0.002**	1.83(-0.62, 3.05)	**0.003**	-0.99(-6.64, 4.66)	0.73	0.08(-0.93, 1.1)	0.87	-0.88(-1.79, 0.03)	0.06	1.08(-1.32, 3.47)	0.38	-0.38(-0.79, 1.54)	0.53	-0.16(-0.37, 0.04)	0.12
	Female	-1.96(-3.2, 0.72)	**0.003**	-1.12(-4.67, 2.43)	0.54	-0.61(-1.61, 0.39)	0.23	-3.81(-8.66. 1.05)	0.12	-0.68(-1.53, 0.17)	0.12	0.64(-0.11, 1.34)	0.09	1.81(-0.38, 3.99)	0.10	-0.47(-0.64, 1.59)	0.40	0.02(-0.13, 0.17)	0.82
**RBDSQ**	Overall	-1.32(-2.5, 0.14)	0.03	1.64(-1.84, 5.11)	0.36	-0.57(-1.49, -0.36)	0.23	-3.48(-7.87, 0.9)	0.12	-0.32(-1.11, 0.47)	0.43	-0.21(-0.95, 0.52)	0.56	0.12(-1.82, 2.07)	0.90	0.17(-0.77, 1.15)	0.72	-0.1(-0.26, 0.07)	0.24
	Male	-0.004(-1.36, 1.35)	0.10	9.64(4.34, 14.93)	**<0.001**	1.13(-0.26, 2.52)	0.11	0.57(-5.83, 6.98)	0.86	-0.21(-1.36, 0.93)	0.71	-0.86(-1.89, 0.16)	0.10	1.85(-0.88, 4.57)	0.18	1.2(-0.13, 2.52)	0.08	-0.29(-0.51, -0.07	**0.01**
	Female	-2.2(-3.7, 0.69)	**0.005**	-3.48(-8.26, 1.3)	0.15	-1.12(-2.47, 0.22)	0.19	-8.74(-15.23, 2.25)	**0.01**	-0.47(-1.59, 0.65)	0.41	0.22(-0.72, 1.16)	0.64	-0.44(-3.37, 2.5)	0.77	-0.54(-2.05, 0.96)	0.48	0.05(-0.15, 0.25)	0.63
**Constipation*RBDSQ**	Overall	0.33(-1.16, 1.81)	0.65	-6.2(-9.87, -2.53)	**0.001**	-0.25(-1.22, 0.73)	0.62	-0.45(-4.27, 5.16)	0.85	-0.01(-0.86, 0.87)	0.99	-0.1(-0.96, 0.76)	0.82	-1.8(-3.92, -0.32)	0.10	-0.73(-1.74, 0.27)	0.15	0.15(-0.06, 0.36)	0.15
	Male	-1.35(-2.85, 0.16)	0.08	-14.61(-20.15, -9.07)	**<0.001**	-2.25(-3.71, 0.79)	**0.003**	-3.99(-10.81, 2.83)	0.25	0.4(-1.64, 0.84)	0.52	0.65(-0.46, 1.76)	0.25	-3.35(-6.24, 0.46)	0.02	-1.68(-3.08, 0.28)	0.02	0.36(0.09, 0.63)	**0.01**
	Female	1.81(-0.05, 3.57)	0.04	0.73(-5.84, 4.38)	0.78	0.79(-0.65, 2.22)	0.28	6.24(-0.7, 13.19)	0.08	0.62(-0.59, 1.82)	0.32	-0.77(-1.84, 0.3)	0.16	-1.6(-4.74, 1.53)	0.32	-0.2(-1.8, 1.4)	0.81	-0.03(-0.25, 0.19)	0.79

The effects of constipation and RBDSQ on cognitive outcomes over 5 years excluding PD subjects with MoCA<26 were analysed using linear mixed-effects modelling adjusted for 1: age and LEDD; 2: age, years of education and LEDD based on gender and adjusted for gender when analysing the overall cohort. In each model, presence/absence of constipation, RBDSQ, constipation*RBDSQ interactive term are the independent variables whereas the neuropsychological test scores are the dependent variables.

Abbreviations

β: β-coefficient (95% CI); HVLT-R: Hopkins Verbal Learning Test-Revised; JLO: Benton Judgement of Line Orientation; LEDD: Levodopa equivalent daily dose; LNS: Letter Number Sequencing; MoCA: Montreal Cognitive Assessment; RBDSQ: Rapid eye movement sleep behaviour disorder questionnaire; SDMT: Symbol Digit Modalities Test

***Supplementary Figure S1*. Receiver operating characteristics (ROC) curves of cognitive impairment at year 5 follow-up visit in PD subjects**

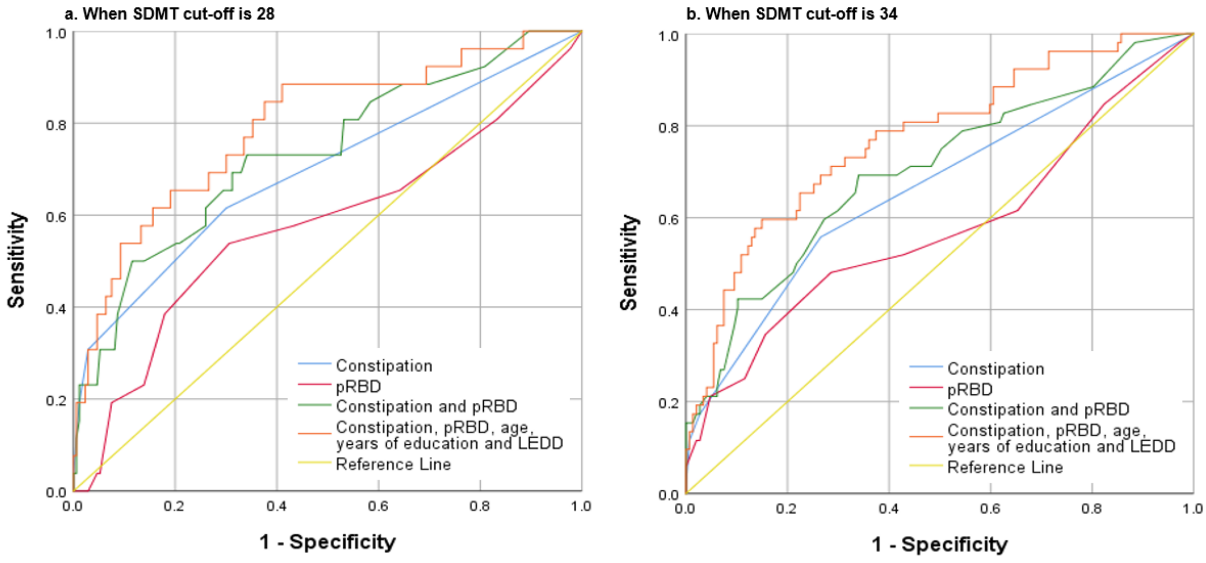

Predictive value of constipation and pRBD with demographic variables (age, years of education and LEDD) was assessed using the ROC curves based on a cut-off score of (a) 28 for SDMT and (b) 34 for SDMT. The AUC values were constipation=0.64, pRBD=0.61, constipation and pRBD=0.73, constipation, pRBD, age, years of eduation and LEDD=0.79 in (a) whereas constipation=0.61, pRBD=0.53, constipation and pRBD=0.70, constipation, pRBD, age, years of eduation and LEDD=0.77 in (b).

Abbreviations

LEDD: Levodopa equivalent daily dose; pRBD: probable rapid eye movement sleep behaviour disorder
